# Experiences of Stroke Survivors and Clinicians With a Fully Immersive Virtual Reality Treadmill Exergame for Stroke Rehabilitation: A Qualitative Pilot Study

**DOI:** 10.3389/fnagi.2021.735251

**Published:** 2021-11-02

**Authors:** Merete Endresen Moan, Elise Klæbo Vonstad, Xiaomeng Su, Beatrix Vereijken, Marit Solbjør, Nina Skjæret-Maroni

**Affiliations:** ^1^Sunnaas Rehabilitation Hospital, Nesodden, Norway; ^2^Department of Computer Science, Faculty of Information Technology and Electrical Engineering, Norwegian University of Science and Technology, Trondheim, Norway; ^3^Department of Neuromedicine and Movement Science, Faculty of Medicine and Health Sciences, Norwegian University of Science and Technology, Trondheim, Norway; ^4^Department of Public Health and Nursing, Norwegian University of Science and Technology, Trondheim, Norway

**Keywords:** stroke, virtual reality, treadmill walking, rehabilitation, exergames

## Abstract

Use of VR-games is considered a promising treatment approach in stroke rehabilitation. However, there is little knowledge on the use and expectations of patients and health professionals regarding the use of treadmill walking in a fully immersive virtual environment as a rehabilitation tool for gait training for stroke survivors. The objectives of the current study were to determine whether stroke survivors can use fully immersive VR utilizing modern HMDs while walking on a treadmill without adverse effects, and to investigate the experiences of stroke survivors and clinicians after testing with focus on acceptability and potential utilization in rehabilitation. A qualitative research design with semi-structured interviews was used to collect data. Five stroke survivors and five clinicians participated in the study and tested a custom-made VR-game on the treadmill before participating in individual semi-structured interview. Data were analyzed through thematic analysis. The analysis of the interview data identified two main categories: (1) *experiencing acceptability through safety and motivation*, and (2) *implementing fully immersive VR in rehabilitation*. Both stroke survivors' and clinicians enjoyed the treadmill-based VR-game and felt safe when using it. The stroke survivors experienced motivation for exercising and achievement by fulfilling tasks during the gaming session as the VR-game was engaging. The clinicians found additional motivation by competing in the game. Both groups saw a potential for use in gait rehabilitation after stroke, on the premise of individual adaptation to each patient's needs, and the technology being easy to use. The findings from this qualitative study suggest that a fully immersive treadmill-based VR-game is acceptable and potentially useful as part of gait rehabilitation after stroke, as it was positively received by both stroke survivors and clinicians working within stroke rehabilitation. The participants reported that they experienced motivation in the game through safety, engagement and achievement. They also saw the potential of implementing such a setup in their own rehabilitation setting. Elements that enable safety and engaging experience are important to maintain when using a fully immersive VR-game in stroke rehabilitation.

## Introduction

Each year, 15 million people world-wide are affected by a stroke, making it the second most frequent cause of death (Ngandu et al., 2015) and the leading causes of disability in adults (Organization, 2003; Feigin et al., 2014). At the same time, there is a decline in stroke mortality due to better care and follow-up (Sarti et al., 2003), leading to an increase in stroke survivors that face impairments in physical, psychological and cognitive functions after the stroke (Engstad et al., 2012; Feigin et al., 2015). The incidence of stroke increases with age in both men and women, with ~50% of all strokes occurring in people over the age of 75 and 30% over the age of 85 (Lui and Nguyen, 2018).

Loss of motor functions in part of the body is one of the most common impairments caused by stroke (Langhorne et al., 2011). In stroke survivors, regaining gait function is the most frequent self-stated rehabilitation goal (Bohannon et al., 1988; Maclean et al., 2000) and plays a vital role in regaining independence (Mant and Walker, 2011). Gait rehabilitation is typically performed as over ground walking or treadmill walking (Park et al., 2013). Treadmill walking is an attractive option as it allows for continuous walking of varying lengths in a safe and controlled environment. Moreover, working with repetitive tasks and adherence to training is essential to increase the amount of training to the levels necessary to regain function after a stroke (French et al., 2016). Likewise, getting immediate response and feedback on the performance is vital for the rehabilitation progress (Schmid et al., 2016).

Previous studies suggest that technology-based rehabilitation, such as serious games in virtual reality (VR), can be effective in rehabilitation after a stroke (Deutsch and Mirelman, 2007; Kim et al., 2011; Laver et al., 2017; Faria et al., 2018). VR is a computer-simulated environment imitating a physical presence in real or imagined worlds where one controls a visualization of one's body to perform different tasks (Howard, 2017). Here, one can improve both physical and cognitive function at the same time, as well as work with repetitive tasks and receive immediate feedback on activity performance. In the western world, VR and interactive video gaming have emerged as treatment approaches, and commercial gaming consoles are being adopted rapidly in clinical settings, also in stroke rehabilitation (Laver et al., 2017). Several studies have designed and tested VR-based systems for rehabilitation of individuals in the chronic phase post-stroke, but the focus has been mainly on upper extremity rehabilitation (Jang et al., 2005; Henderson et al., 2007). Although not as extensive as the upper extremity work, there have also been efforts to design and test VR systems to improve walking ability of people post-stroke (Jaffe et al., 2004; You et al., 2005; Fung et al., 2006; Mirelman et al., 2010), and VR-based intervention has been found to have advantage in improving balance training, gait speed and the quality of gait, compared to conventional interventions (Corbetta et al., 2015; Darekar et al., 2015).

In recent years, advances in technology have made it possible to provide a *fully immersive* VR-experience by using head-mounted displays (HMDs). This way, the wearer is visually and potentially audibly separated from the real environment (Waller and Hodgson, 2013). It has been suggested that HMD-based games are likely to increase the sense of presence, to be more engaging, and thereby have even greater potential for motor (re-)learning than less immersive forms of VR, such as desktop-based and cave automatic virtual environments (CAVE), (Guo et al., 2013). Particularly, the enriched environment and goal-oriented tasks with repetition that are possible in fully immersive VR stimulate the neuroplasticity of the injured brain in terms of interest and motivation which is important in rehabilitation after stroke (Levin, 2011). Immersive virtual reality extends the affordances of merely a screen, allowing the user to enter a different world surrounding them in three dimensions. This allows for interaction with elements while performing novel functions that might not be as easily achieved in traditional rehabilitation settings (Blascovich et al., 2002; Patel et al., 2006). However, evidence supporting the use of fully immersive VR in gait rehabilitation is largely lacking at the moment, as very few studies so far have focused on *fully immersive* VR for gait rehabilitation. Jaffe et al. (2004) designed a system using HMDs while walking at a self-selected speed on a treadmill secured with a harness. They found that virtual objects were just as effective as real objects in shaping the stepping characteristics of individuals with post-stroke hemiplegia. Likewise, Kim et al. (2017) found that both healthy older adults and people with Parkinson's disease were able to successfully use fully immersive VR during walking without adverse events. However, a fully immersive VR treadmill exergame can expose the individual to tasks beyond merely viewing a scene as in the study by Kim et al. (2017). A good example of this is the VR environment used by Winter et al. (2021) where players rebuild their companions world by walking on a treadmill. The technology can easily be used to create a multi-component environment where the player can perform more complicated tasks that focus on training specific functions while walking on a treadmill—although these could turn out to be too demanding and cause frustration or rejection (Laver et al., 2015). However, previous studies using VR for upper extremity training after stroke, found that both stroke patients and clinicians enjoyed using VR, valued the intensive and motivational character of VR training, and saw VR as an opportunity to participate in enjoyable activities bridging environmental or psychological barriers without serious adverse effects (Farrow and Reid, 2004; Lewis et al., 2011; Schmid et al., 2016). Also, Cortés-Pérez et al. (2020) found that immersive VR had higher effects on improving balance and reducing the risk of falls than conventional therapy in their study that aimed to assess whether an experimental protocol based on immersive VR therapy was valid for stroke rehabilitation. Clinicians also enjoy seeing patients' activity, engagement and motivation for activity (Schmid et al., 2016), which indicates that clinicians perceive VR as a complement to conventional methods for upper extremity rehabilitation after stroke as long as the training is guided by motor learning principles (Schmid et al., 2016). Also, walking on a treadmill using VR has been found feasible and acceptable by people suffering a stroke (Winter et al., 2021). These earlier studies provide promising indications that fully immersive VR on a treadmill may be applicable to rehabilitation after stroke. However, it has been emphasized that VR cannot replace the therapist's clinical reasoning or their social interaction with the patients (Schmid et al., 2016). If fully immersive VR is to be implemented widely as part of stroke rehabilitation, more knowledge is needed about the feasibility and acceptability of use by gaining insight from the experience of use from stroke survivors and health professionals working within rehabilitation. The current study explores stroke survivors' and clinicians' initial experiences of testing a fully immersive VR exergame on a treadmill for gait rehabilitation after stroke. Specifically, the objectives of this study were to determine whether stroke survivors found the fully immersive VR utilizing HMDs while walking on a treadmill acceptable without experiencing adverse effects, and to investigate the user experiences of stroke survivors and clinicians after testing with focus on potential utilization in rehabilitation after stroke.

## Materials and Methods

### VR-Game

**Figure 1:**
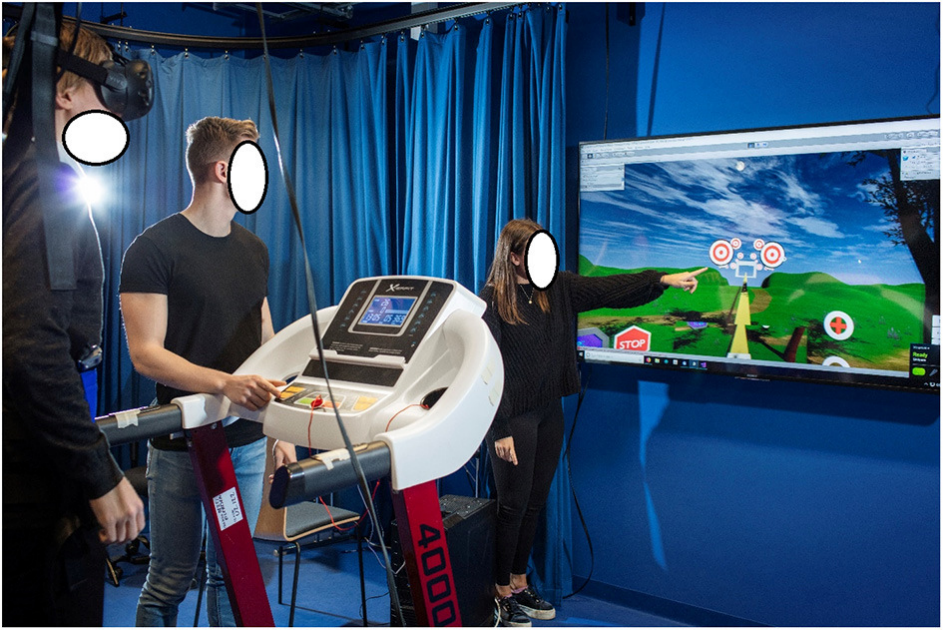
VR-mill game setup. The player is wearing an HMD and a safety harness while walking on the treadmill. Clinicians or assistants can see a 2D image of the scene and the player's actions on the screen in front of the treadmill.

The game used in this study was a prototype of a treadmill-based Virtual Reality exergame developed by 3D-Motech called “*VR Mølle*” (“VR-Mill”) [Unity, version 18.4], (Figure 1). The game uses a Virtual Reality headset (HTC Vive), an off-the-shelf treadmill (X-erfit 4,000 Pro Runner), and a 3D motion capture system (Qualisys AB, Gothenburg, Sweden).

The exergame is played by walking on a treadmill while performing cognitive exercises visualized in a VR headset. The player's movements are tracked by a 3D motion capture system through reflective markers attached to the feet. The player's feet are continuously displayed in the virtual world. The speed in the VR world is synchronized to the speed of the treadmill and can be adjusted during the game. The railing of the treadmill is visible in the game and can be used as support if needed. Moveable Lite Gait harness was installed to secure the player in case of a balance disturbance or fall.

**Figure 2:**
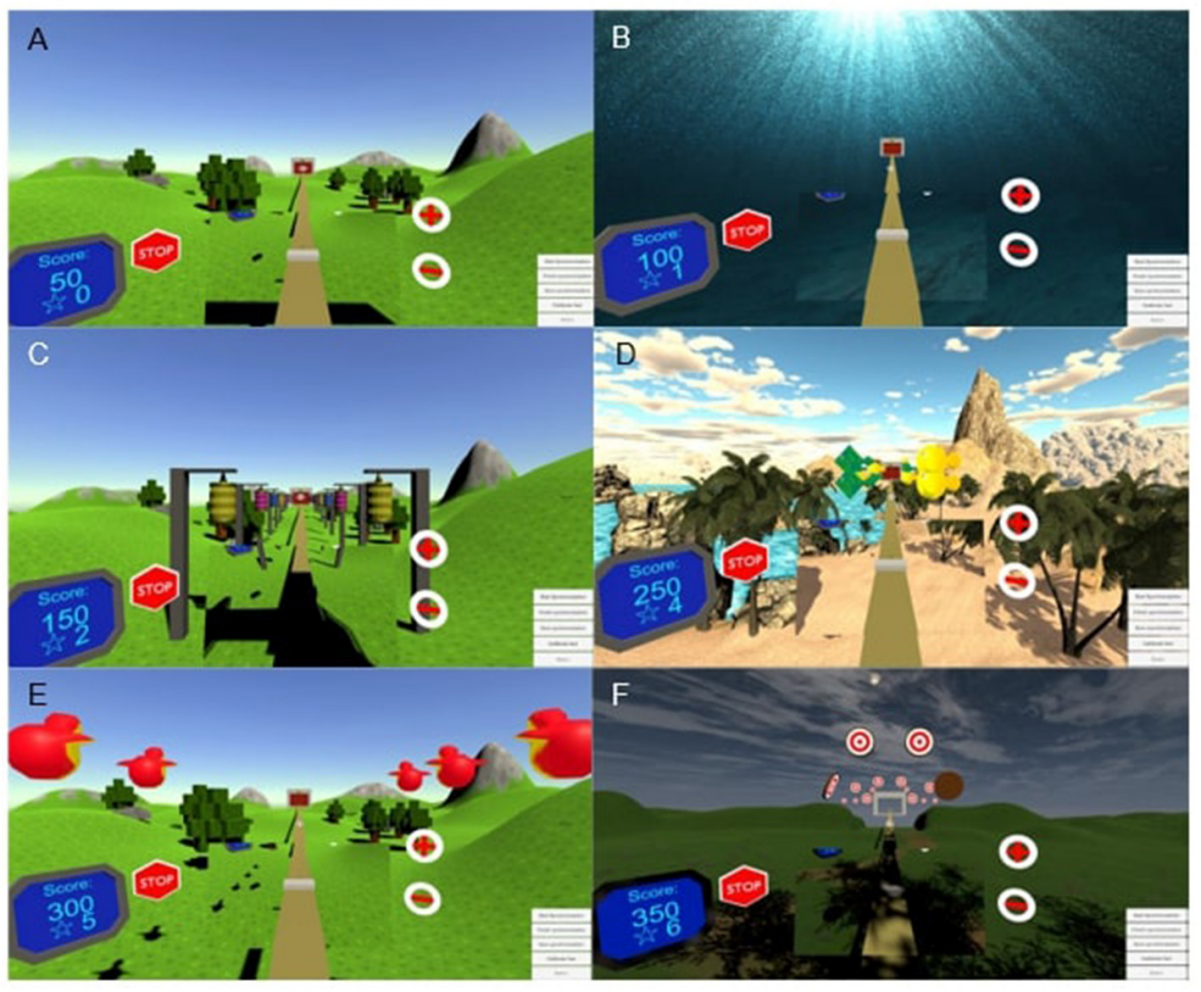
**(A–F)** Screenshots from all six mini-games.

The exergame consisted of six mini-games (Figure 2). The first two mini-games are introduction worlds to familiarize the user with walking on the treadmill while wearing the VR-equipment and setting the user-preferred treadmill speed for the exercise session (Figures 2A,B). The next three mini-games (Figures 2C–E) add different tasks where the user must aim at targets using head movements while walking. By completing these tasks, the user earns stars to be used for shooting at targets in the 6th and final mini-game (Figure 2F). The final mini-game was performed standing still and was not included as a part of the exercise in this experiment. The total walking distance was fixed at 620 m (100 m in each mini game, with 10 m walking before and after the entire game).

In the current study, the VR-game was played individually in a visualization and motion capture laboratory. A researcher, an assistant and a computer technician were present during the use of the VR-Mill. All participants played the six mini games on the VR-Mill once. As the game is set to each player's preferred gait speed, the playing time varies from person to person. Before gameplay, the treadmill system and exergame were demonstrated and explained to the participants. All participants were fitted with a safety harness (Lite Gait, USA) and the VR-headset, and the built-in safety features within the treadmill were demonstrated. These included user access to an emergency stop button and speed controls within the exergame (as illustrated in Figure 2). Virtual representations of the treadmill position gave the participants the possibility to locate and hold on to the treadmill handrails during the exergame.

### Study Design

This was a qualitative interview study which instigated experiences with the VR-game among stroke survivors and clinicians working with stroke rehabilitation. Study participants performed the VR-game on the treadmill (as described above) before participating in a semi-structured interview at the venue of the VR-lab.

### Recruitment

Two groups of individuals were recruited to the study. Stroke survivors were included in the study if the stroke had occurred more than 6 months before data collection, they were able to walk safely without a walking aid, could follow instructions, and had not been diagnosed with epilepsy, aphasia, or neglect. Clinicians who were included had to be employed in stroke rehabilitation at the time of data collection. All individuals were recruited through a rehabilitation center in Central Norway. Clinicians were invited to the study at their place of work, and the stroke survivors were contacted at the rehabilitation center during their stay or received a phone call from one of the employees at the center. Of the ten individuals who opted into the study, five clinicians and three of the stroke survivors received the information letter by mail and the two remaining stroke survivors received it directly from an employee at the rehabilitation center. Time and date for the try-out and the interview was planned individually with each participant. All study participants provided written consent prior to participating in the study.

### Participants

Five stroke survivors and five clinicians participated in the study. Of the five participants that had suffered a stroke (mean age 59, range 40–75 years), two were women. Two stroke survivors had suffered an ischemic stroke, two a hemorrhagic stroke, and one had a combination of both types. Three had an affected right side where two had drop foot, and two had an affected left side. The five clinicians (mean age 42.8, range 36–48 years) consisted of four women and one man. Two were physiotherapists, two were occupational therapists, and the last was a speech therapist. Their work experience within stroke rehabilitation ranged from 2.5 to 20 years, with a mean of 8.4 years. None of the participants in the study had previous experience with VR-games, but all had experience from using a treadmill.

### Data Collection and Analysis

Interviews were carried out in a separate room at the VR-lab during October 2018 to January 2019 by the first author (MEM). Each interview lasted from 18 to 43 min. Interviews were based on a semi-structured interview guide for each group of participants. Each interview guide was developed to investigate how participants experienced the use of the VR-Mill. Both interview guides focused on users' perspectives on safety, motivation, and potential adoption in their rehabilitation process. The development of each interview guide was based on theories on motivation (Deci and Ryan, 1985; Lillemyr, 2007). In addition, the clinicians' interview guide included questions on the potential for stroke rehabilitation and implementation (see Table A1). The first author (MEM) used the interview guide as a flexible framework for the structure of the interview. The interview guide had suggestions for follow-up questions if needed. All the interviews were audio-recorded and transcribed verbatim.

Data analysis was guided by reflexive thematic analysis which is described by Braun and Clarke (2020) as a flexible approach which could be used for experiential qualitative research. It allowed us to explore experiences of participants who were first time users of a treadmill VR-game designed for rehabilitation use, which meant that the experience was not part of their lifeworld but rather an experiment for the participants. Moreover, we chose reflexive thematic analysis since it is not resting upon one theoretical stand but allows the use of theory at several levels of the analysis (Braun and Clarke, 2020).

Reflexive thematic analysis includes a six-step process identifying prominent themes (Braun and Clarke, 2013). In the first step, the aim is to get an overview of, and get to know the data material (Braun and Clarke, 2013). The first author (MEM) read through all the transcribed interviews several times, marked paragraphs and took notes relevant for the following coding process. Three other authors (BV, MS, NS-M) read and took notes from a sample of the interviews and contributed to all stages of the analysis. One author (MS) has substantial competence, and all other authors some competence, in qualitative analysis, which has contributed to the validity of the results. The second step of the analysis is to generate initial codes. The transcribed interviews were read in detail and the material was coded. Codes were compared between interviews in order to get a sense of the whole, understand connections between the codes and to construct categories, main themes and sub themes (see Table 1). All authors participated in discussions about coding. In the next step, the aim was to systematize the data material under different themes (Braun and Clarke, 2013). During this step, we separated quotes from each of the two participant groups into two documents and examined for patterns across participants and variance between participants. To construct a theme, the codes had to have common traits and enlighten the theme from different perspectives. One example of codes for the theme *safety* was *safety harness, handrails, familiar equipment, no experience of dizziness, safe experience* (see Table 1). Step four, reviewing themes, consisted of refining the themes that was made in step three (Braun and Clarke, 2013). To do this, the first author made two mind maps to get an overview of all the themes. These were discussed by all authors to reach consensus on the valid constructions of each theme (see Table 1), and in defining and naming themes (Braun and Clarke, 2013). The initial codes “experiences with treadmill training,” “safety harness,” “handrails,” “no experience of dizziness” and “safe experience” were grouped as the theme *safety*. The initial codes “fun game,” “a feeling of being in the game,” “new technology” and “collecting points” were grouped as the theme *motivation*. Initial codes concerning *implementing VR* were “cognitive function,” “physical function,” “goals for rehabilitation,” “changes and improvement,” and “when to use the VR-game in rehabilitation” (see Table 1). Subsequently, our analysis resulted in two main categories answering our objectives in the current study: (1) *experiencing acceptability through safety and motivation*, and (2) *implementing immersive VR in rehabilitation*. Category 1 consist of the subcategories “safety harness and handrails,” “familiarity with the technology,” “having fun” and “getting feedback.” In category 2 the subcategories are “conditions relating to patient health,” “training multiple functions simultaneously,” “adaptation to individual users” and “organizational difficulties.” The names of the subcategories are based on the initial codes in Table 1. In Table 2 selected quotes from the data material are presented under each subcategory to exemplify the analytical findings (see Table 2).

**Table 1:** Coding process of the reflexive thematic analysis.

**Initial codes**	**Sub themes**	**Main themes**
Safety harnessHandrailsNo experience of dizzinessSafe experience	Safety	Experiencing acceptability through safety and motivation
Fun gameA feeling of being in the gameNew technologyCollecting points	Motivation	
Cognitive functionPhysical functionGoals for rehabilitationChanges and improvementsWhen to use the VR-game in rehabilitation	Implementing VR	Implementing VR in rehabilitation

**Table 2:** Quotes to exemplify the analytical findings.

**Categories**	**Subcategories**	**Quote number**	**Quotes**	**Anonymous identification**
Experiencing acceptability through safety and motivation	Safety harness and handrails	*Quote 1*	“*. I was held up by the safety harness, then you didn't have to worry about falling down.”*	Ron 75 years, person with stroke
	Familiarity with the technology	*Quote 2*	“*We use safety harness at [the rehabilitation center], so some of the equipment is familiar.”*	Andy 37 years, clinician
	Having fun	*Quote 3*	“*That went by terribly fast when I think about the fact that I walked 620 meters. You hardly think about the walking. That was great, I thought it was fun.”*	Miriam 64 years, person with stroke
		*Quote 4*	“*It's fun because it's new and different. You want to master those tasks, it's fun when you master it.”*	*Robert 48 years, clinician*
	Getting feedback	*Quote 5*	“*It was fun collecting points and see that you could master the tasks in the game.”*	Kate 53 years, person with stroke
Implementing immersive VR in rehabilitation	Conditions relating to patient health	*Quote 6*	“*There is a lot of cognitive training. need to pay attention to what's going on, the tempo and concentration and focus, a lot of those things get stimulated.”*	Robert 48 years, clinician
	Training multiple functions simultaneously	*Quote 7*	“*. you need to use your head as early as possible, you know, concentrate. It's something entirely different than being at [the rehabilitation center], where they almost do your thinking for you, but, like the VR-game is something entirely different.”*	Jim 48 years, person with stroke
		*Quote 8*	“*. choose different levels… you achieve one level, and maybe manage to get to the next level.”*	Kate 53 years, person with stroke
	Adaptations to individual users	*Quote 9*	“*If I could increase or decrease the difficulty level… tailored to the person's skills, then it would be very useful.”*	Andy 37 years, clinician
	Organizational difficulties	*Quote 10*	“*… if it takes too long to get the VR-game ready, the required effort exceeds the benefits … it's an important factor that the game doesn't glitch.”*	Julia 41 years, clinician

### Ethics

The study was approved by the Norwegian Center for Research Data and the Regional Ethical Committee for Medical and Health Research Ethics and conducted in accordance with the Declaration of Helsinki. Informed written and verbal consent to participate were obtained from the participants. Participation was voluntary, and withdrawal was possible at any time without changes to ongoing or future rights to treatment. All information was processed without name or personal identification number, or any other information that was directly identifiable of participants. To preserve anonymity, identifiable places or situations related to the participants have not been provided in the article. Audio files were stored according to the ethical approval.

## Results

All participants completed the mini games without adverse events. The stroke survivors used a mean of 21.45 (range 18.11–31.39 min) min to complete the five-exercise mini-games, while the clinicians used a mean of 10.6 (range 9.5–15.33 min) min.

### Experiencing Acceptability Through Safety and Motivation

Before the treadmill testing started, some of the stroke survivors had been concerned about whether they would be able to play the game, as they had seen younger people play games that looked too difficult for themselves. The participants' lack of experience with VR games led them to feeling unprepared for what they were about to do. However, after experiencing the VR-game, they expressed engagement and joy from participating. Receiving instructions prior to playing, and reminders about what was to come next while playing, contributed to mastering the game. No one experienced dizziness or motion sickness while playing, and the VR-headset did not hinder their movements.

### Safety Harness and Handrails

The stroke survivors and the clinicians identified two common elements that contributed to the experience of safety: the harness and the handrails. When having these two elements, the stroke survivors felt safe and were not concerned about falling or injuring themselves (Table 2, quote 1). The clinicians also liked to know that they could hold on to the handrails whenever they wanted.

### Familiarity With the Technology

A third element that contributed to safety for the stroke survivors was being familiar with using treadmills and having used treadmills in previous exercise and rehabilitation. The clinicians also focused on the control buttons visible in the VR-environment, which provided the possibility to stop playing when feeling uncomfortable. The opportunity to stop the game while playing could, according to the clinicians, lower the bar to try the game for persons that might feel unsure about this type of technology (Table 2, quote 2).

### Having Fun

For the clinicians, the VR-game was perceived as new, exciting, and different from conventional rehabilitation, thereby holding the potential to motivate persons with stroke who are less motivated for traditional rehabilitation. They saw the game as motivating and fun to play, while at the same time having the potential to provide good quality exercise of gait function. For the stroke survivors, the VR-system provided a dynamic setting, and the mini games were fun to do while walking on the treadmill. Both the stroke survivors and the clinicians reported experiences of forgetting that they were walking on a treadmill or even forgetting themselves. For some of the stroke survivors, it was a surprise to learn that they had walked for a longer distance and longer duration than they had expected. This contributed to motivation and engagement (Table 2, quote 3).

Forgetting time and space was regarded as very positive by the clinicians as well, especially as it was experienced while exercising. Their stories contained feelings of being a part of the game and being in a different dimension. They described this as being in a state of flow, which they saw as a source of excitement, motivation, and joy for themselves as well as for their patients (Table 2, quote 4).

### Getting Feedback

Visualization, such as lights that lit up when they hit the target, provided immediate feedback throughout the game. Some of the persons with stroke experienced the collection of points as a motivational factor. They also experienced immediate feedback as motivating, especially through the built-in reward system of collecting points. One of the stroke survivors could relate the last mini game to earlier experiences of shooting clay pigeons. Motivation increased even more when hitting the targets in the last mini game.

The competitive element of the game was motivating for the clinicians. They enjoyed competing with themselves (or others) to increase their scores. Clinicians expected that collecting points to use in the last mini game could motivate patients as future users of this game. An element that would motivate clinicians toward using this technology was the opportunity to measure progress during the rehabilitation process. The game's social aspect was also appreciated as it could lead to new conversations if users wished to share their experiences with friends and family (Table 2, quote 5).

### Implementing Immersive VR in Rehabilitation

All participants in the study found the VR-game to be beneficial for implementation in rehabilitation services but highlighted the need to adapt specific play options to specific patients.

#### Conditions Relating to Patient Health

The stroke survivors felt good after completing the VR-game and thought that the game should be implemented in rehabilitation if cognitive and/or functional impairments were present. However, in their opinion, users would need a certain level of physical and cognitive functioning to master the game while walking. This was also pointed out by the clinicians. Abilities such as attention, concentration, memory, and the capability to plan what to do next were important, as one needs to pay attention to the tasks in the game while planning the next action. A possible obstacle of using the game system in the early stages of the rehabilitation process could be lack of concentration, dizziness, or wandering thoughts (Table 2, quote 6).

For the stroke survivors, challenges with concentration, attention, and memory while simultaneously walking was a new experience within rehabilitation exercises, and they found the VR-game to be an effective way to exercise.

#### Training Multiple Functions Simultaneously

Previous rehabilitation programs had typically targeted one element of physical or cognitive functioning at the time. During their own previous rehabilitation process, the clinicians had controlled the exercise and progress, but the VR-game allowed them independence, through challenging their concentration, attention and memory. The stroke survivors could see themselves using the game several times per week as part of their post-acute rehabilitation. They described the VR-game as different and exciting, which was important regarding variation and motivation during a rehabilitation process (Table 2, quote 7).

The stroke survivors gave feedback about possible changes and suggestions that could further improve the usefulness of the VR-game, such as training reaction time and adapting the difficulty level depending on the user's level of functioning. One suggestion was to avoid repetition with two similar worlds at the beginning of the game. Another suggestion was to implement more tasks in the mini games (depicted in Figure 2) to allow participants to reach additional difficulty levels (Table 2, quote 8).

#### Adaptation to Individual Users

Both the stroke survivors and clinicians indicated that it should be possible to tailor the game to each user to adapt to each player's individual level of functioning. Clinicians stated that they would have liked the opportunity to ensure that the exercise is in line with each rehabilitation program's goals and patients' goals. If the game could be adapted to different rehabilitation needs, the game would be relevant for even more patient groups and appropriate for a longer period of the rehabilitation stay. This could also justify a higher cost when implementing the game in a rehabilitation center. Furthermore, including tasks that resembled real-life tasks, such as stepping over objects, could also add relevance to the game. The clinicians thought this could increase balance training in the mini games (Table 2, quote 9).

#### Organizational Difficulties

Some of the clinicians described that having an VR-game like this at the rehabilitation center would be a dream, as they felt that VR-games like the one used in this study, could be useful for many of their patients, and could be combined with conventional rehabilitation. However, implementation would necessitate the possibility of adjustments and removing potential barriers to using the technology, such as experiencing problems with the system's technical components or using time to get the system ready. Clinicians did not want to risk wasting valuable time on dealing with technical issues that they could otherwise devote to their patients through conventional therapy (Table 2, quote 10).

## Discussion

This study explored acceptability and potential utilization of a fully immersive VR-game on a treadmill for gait rehabilitation after stroke through testing and interviews with stroke survivors and clinicians working in stroke rehabilitation. To the best of our knowledge, this is the first study to investigate both clinicians' and stroke survivors' perspectives regarding a fully immersive gait-specific VR-game. Analysis of the interviews identified two key categories of considerations for using VR-technology in stroke rehabilitation: experiencing acceptability through safety and motivation, as well as implementing immersive VR in rehabilitation. Both stroke survivors and clinicians enjoyed trying the VR-Mill game and felt safe when using it. The stroke survivors experienced motivation for exercise during the treadmill session as the VR-game was engaging, as well as experiencing achievement when fulfilling tasks during the game. The clinicians found additional motivation by competing in the game. Both groups saw a potential for use in gait rehabilitation after stroke, as long as the game could be adapted to each individual user and the technology would be easy to use.

Feeling safe during rehabilitation is of paramount importance for a person after stroke and needs to be addressed adequately (Laver et al., 2017). When using VR in neurological patients, visual or cognitive overload must always be avoided as this might act as a confounding factor (Lewis and Griffin, 1997). If being or feeling unsafe, the risk of falling and the fear of falling may significantly hamper the use and potential benefit of VR exercises. Despite the ample focus on the use of VR in stroke rehabilitation in recent years (e.g., De Rooij et al., 2016; Porras et al., 2018), there is sparse knowledge on the safety of using fully immersive VR games over a longer duration of time. Results in our study revealed that stroke survivors felt safe when walking on the VR-Mill for over 20 min and there were no adverse events nor any cases of motion sickness or dizziness. A safety harness, use of handrails and being familiar with walking on a treadmill were all highlighted as being important for feeling safe. The sense of control provided by the possibility to directly pause and stop the game was also mentioned as critical for feeling safe. Participants in the current study did not report any difficulties in understanding the tasks or comprehending the virtual world. This might be due to the game and virtual environment being specifically developed for this patient group to avoid confusion and misinterpretation while playing. Furthermore, the fact that the belt on the VR-Mill moved at the same speed as the virtual world may have prevented the motion sickness that is often experienced when there is a mismatch between visual and motor-sensory stimuli (Hettinger and Riccio, 1992). Similar results were found in a study from 2011, where older adults did not experience motion sickness or dizziness after using an HMD while walking on a treadmill (Parijat and Lockhart, 2011). Our findings are also in line with other research on motion sickness from using HMDs (Huygelier et al., 2019), although there might be some bias in our results, since the exposure time was relatively short, and increased exposure time increases the risk for experiencing motion sickness from HMD use (Duzmańska et al., 2018). The participants in this study did not report any difficulties in understanding the tasks or comprehending the virtual world. This might be due to the game and virtual environment being specifically developed for this patient group to avoid confusion, cyber sickness and misinterpretation while playing.

Motivation is seen as an important factor in adherence to the rehabilitation process as people with stroke have been found to be more likely to understand the nature and purpose of their rehabilitation when having high motivation (Maclean et al., 2000). In the present study, both clinicians and stroke survivors enjoyed playing the game, which is in line with previous research that confirms “fun experience” as a motivating effect of VR-based rehabilitation (Pallesen et al., 2018). Mastering the game, despite their lack of VR-experience, contributed highly to motivation for continuing the gameplay. While playing, the participants experienced a state of flow which was tightly connected to their enjoyment. Although previous studies have hypothesized that the sense of presence is stronger in an immersive environment than that in a non-immersive environment (Ventura et al., 2019), it is not clear whether reaching the flow zone is a consequence of the immersive nature of the exergame or if the immersive nature of the game has in any way impacted the state of flow. A controlled experiment where this is compared with the results from a non-immersive exergame is needed to understand this, which could be an interesting future study as we now have established the feasibility of such a setup. Another possible contributing factor is that the activities in the mini-games were goal-directed, which required concentration and appropriate cognitive and physical skills, which in turn made the tasks challenging yet attainable (Sweetser and Wyeth, 2005). It can be hypothesized from our study that an immersive approach that does not exceed the stroke survivors' skills, combined with the design of the mini-games (challenging activities with clear goals and immediate feedback that require skills and concentration) led participants into the flow zone. Using this type of technology is potentially very useful as part of stroke rehabilitation as it provides intensive, task-specific, multi-component training. The combination of VR and treadmill exercise can help to increase training volume and the courage and motivation to exercise for a more extended period (Sisto et al., 2002; Fung et al., 2006), which are essential to regain function after stroke. This was also seen as one of the main benefits of the system by the participants, especially the clinicians. The immediate feedback on performance was also considered encouraging and contributed to motivation, which was also shown in an earlier study by Kim et al. (2011). Furthermore, using fully immersive HMD-based VR on a treadmill provides new opportunities for visual independence from the support surface, which is highlighted as an important quality of adaptive behavior (Mulder et al., 2002). For example, in the three mini games C to E, the participants need to turn their heads and eye gaze in order to hit targets in the game. The headset was able to capture the head movements and only provided rewards when the head was turned properly, indicating that the players were able to take their eyes off their feet or support surface without becoming disoriented or falling down (Mulder et al., 2002).

Proper information on use and safety, as well as removing potential barriers related to using the technology itself, need to be addressed adequately to avoid frustration among clinicians and stroke survivors when implementing new technology into health services (Pallesen et al., 2018). The VR-Mill game in this study has a relatively complex setup, which could be an obstacle for its implementation into clinical rehabilitation services, as it is found that characteristics such as difficult set-up, discomfort, and lack of time and knowledge could hinder the use of technology (Levac and Miller, 2013; Nguyen et al., 2018). Furthermore, having individually tailored relevant tasks is vital. Tailoring tasks and adapting the challenge level is seen as a major potential for positive improvement (Levin et al., 2014; Shin et al., 2014). It is essential that the clinicians working within rehabilitation are aware of each patient's strengths and weaknesses (Schmid et al., 2016) and adapt the tasks and difficulty level in the game tasks to each specific rehabilitation goal in order to increase the motor (re)learning and improve the possibilities for a better rehabilitation outcome. One concrete suggestion that was raised in our study was to include balance training elements to increase the training of the lower limbs. As balance control is essential for optimal gait function (Winter, 1995), it would indeed be beneficial to add elements in the game that can be tailored to each individual to challenge postural control even more. Some participants also suggested adding some kind of social interaction in the game, which is considered a contributing factor to motivation and enjoyment (Sweetser and Wyeth, 2005). Finding ways to incorporate social interaction elements is therefore of interest for further development of VR technology.

This study is an initial step toward establishing fully immersive VR-games on a treadmill for gait rehabilitation after stroke. However, there are some limitations that need to be taken into account. First, the included study sample was small which could limit the interpretation of the results. However, this was the first time the VR-Mill was tried with stroke survivor and the provided information on experiences and motivation for further use in rehabilitation after stroke paves the way for testing on a broader scale and possible implementation of the system in a rehabilitation setting to assess effect on rehabilitation outcomes. Further, the study sample was constrained by availability and access to patients, and the participants were motivated and interested in testing such technology, which in turn could influence their feedback of the system toward being more positive. Additionally, the analytical approach with reflexive thematic analysis aims for validity through reflexive discussions of coding and construction of main themes. It could be a limitation to the validity of the results that we did not assess inter-coder reliability. Also, this study is merely based on subjective feedback from the participants and did not include standardized tools to measure acceptance. The fact that the results are linked merely to the first impression of using the system for a very limited amount of time might also be a limitation and an evaluation after sustained use would provide more robust indicators. However, as this was an explorative study where the aim was to gain a broader perspective of the possibilities of use in a rehabilitation setting more than just feedback on the system itself. Lastly, the experimental setting of the VR-Mill testing may have been a limitation compared to a study in a more real-life setting such as a rehabilitation clinic. Future studies should aim toward testing the technology in a larger prospective trial in real rehabilitation settings.

## Conclusion

The current study provides valuable insights into the use of a fully immersive treadmill VR-game for gait rehabilitation after stroke. The game, VR-Mill, was positively received both by stroke survivors and clinicians working within stroke rehabilitation as they found the game to be both acceptable and potentially useful as part of gait rehabilitation after stroke. The results from the current study illustrate that a VR-game on a treadmill can be safe for the players as long as safety measures such as handrails and harnesses are available. Furthermore, using an immersive VR-game is motivating as the participants found it fun and enjoying as well as experiencing a feeling of flow when playing. As both clinicians and stroke survivors saw a high potential of implementing VR-games in clinical practice, future studies should aim to develop and improve the systems such that they can easily be implemented in clinical settings, where use of resources and feasibility can be further studied. In addition to ensure the safety of the players, future studies should also strive to add more quantitative measures of function like the number of steps taken or objects hit while walking on targets that can provide feedback to professionals in clinical settings to help guide rehabilitation. In addition, studies should strive toward gaining a better understanding of the relationship between immersion and motivation and include games that provides sustained motivation for use, preferably over a longer period of time.

## Data Availability Statement

The datasets presented in this article are not readily available because audio files and transcripts are not available for review because of the risk of participant identification. Requests to access the datasets should be directed to nina.skjaret.maroni@ntnu.no.

## Ethics Statement

The studies involving human participants were reviewed and approved by Regional Ethical Committee for Medical and Health Research Ethics, REC Central, Norway. The patients/participants provided their written informed consent to participate in this study.

## Author Contributions

EV, BV, and NS-M conceived the study. MM, BV, and NS-M formulated the research question and designed the qualitative study. MM and MS developed the interview guide and conducted the data analysis. MM and EV conducted the VR-Mill testing and drafted the manuscript. MM interviewed all study participants. All authors contributed to the manuscript text, edited, and approved the final manuscript.

## Conflict of Interest

The authors declare that the research was conducted in the absence of any commercial or financial relationships that could be construed as a potential conflict of interest.

## Publisher's Note

All claims expressed in this article are solely those of the authors and do not necessarily represent those of their affiliated organizations, or those of the publisher, the editors and the reviewers. Any product that may be evaluated in this article, or claim that may be made by its manufacturer, is not guaranteed or endorsed by the publisher.

## Appendix

**Table A1:** Interview guides.

**Interview guide for persons with stroke**	
1.	Could you please start by telling a little bit about yourself? (Age, occupation, your experiences in rehabilitation, earlier life)
2.	Could you tell me about how you experienced trying the VR-Mill? • Could you tell me why or why not you experienced it as safe to use? • Did you experience any form of dizziness or nausea while playing? • Have you ever used a treadmill earlier?
3.	Did you experience the game as meaningful or motivating to use? • Could you please say a bit more about what made the game motivating? • How did you experience the collecting of points during the game?
4.	Was there something lacking in the game that could have made the game more motivating or more fun to use? • Do you have any thoughts on how such games may contribute to motivation to do work outs in everyday life?
5.	What did you like and what did you not like about using the game?
6.	Would you like to use the VR-Mill one or several times more? • Why, why not?
7.	How did you experience the introduction to the VR-Mill ? • How challenging, how easy?
8.	What do you think about this form for training? • For gait function? • For balance?
9.	Could you tell me about your earlier experiences with computer gaming or Virtual Reality? • How did your previous experiences with such technology affect today's testing? Did it (not) affect you?
10.	In your opinion, for whom is this form of rehabilitation suitable (i.e., patients)? For other persons you know, or for other groups?
11.	Before we end the interview: would you like to add something to any of the questions, or are there any important subjects that we haven't touched upon yet? Thank you for your participation.
**Interview guide for clinicians**	
1.	Could you please start by telling a little bit about yourself? (Occupation, work experience, age)
2.	How did you think it was to use the VR-Mill? • Did you experience it as safe? • As meaningful? • As motivating?
3.	How did you experience the introduction to how the VR-Mill works? • How challenging / how easy?
4.	In your opinion, in which way can the VR-Mill be used by persons with stroke with reduced gait function? • Should some features of the VR-Mill or game be changed? • Which elements are most important for the usability of the VR-Mill?
5.	What did you like and what did you not like about the gait training while using the VR-Mill? • Why?
6.	Why or why not would you recommend this training method to your patients?
7.	In which way could the VR-Mill be used at your workplace? • What are the potential positive effects of implementing it? • What are the potential negative effects of implementing it? • Do you see any barriers for implementing it at your workplace?
8.	To which groups of patients would you recommend this form of rehabilitation?
9.	Do you have any previous experiences with VR or computer gaming?
10.	Before we end the interview: would you like to add something to any of the questions, or are there any important subjects that we haven't touched upon yet? Thank you for your participation.
